# *ETV4* plays a role on the primary events during the adenoma-adenocarcinoma progression in colorectal cancer

**DOI:** 10.1186/s12885-021-07857-x

**Published:** 2021-03-01

**Authors:** Aline Simoneti Fonseca, Anelisa Ramão, Matheus Carvalho Bürger, Jorge Estefano Santana de Souza, Dalila Lucíola Zanette, Greice Andreotti de Molfetta, Luiza Ferreira de Araújo, Rafaela de Barros e Lima Bueno, Graziela Moura Aguiar, Jessica Rodrigues Plaça, Cleidson de Pádua Alves, Anemari Ramos Dinarte dos Santos, Daniel Onofre Vidal, Gyl Eanes Barros Silva, Rodrigo Alexandre Panepucci, Fernanda Maris Peria, Omar Feres, José Joaquim Ribeiro da Rocha, Marco Antonio Zago, Wilson Araújo Silva

**Affiliations:** 1 Department of Genetics, Ribeirão Preto Medical School, University of São Paulo, Av Bandeirantes, 3900, CEP: 14049-900, Monte Alegre, Ribeirão Preto, SP Brazil; 2 Center for Cell Based Therapy and National Institute of Science and Technology in Stem Cell and Cell Therapy, Ribeirão Preto, SP Brazil; 3 Center for Integrative Systems Biology - CISBi, NAP/USP, Ribeirão Preto, SP Brazil; 4 Research Institute Pelé Pequeno Príncipe, Av Silva Jardim, 1632, CEP: 80250-060, Água Verde, Curitiba, PR Brazil; 5 Laboratory of Applied Science and Technology in Health (LASTH), Instituto Carlos Chagas, Fundação Oswaldo Cruz, Curitiba, PR Brazil; 6 Laboratory of Immunofluorescence and Electron Microscopy (LIME), Presidente Dutra University Hospital (HUUFMA), São Luís, MA Brazil; 7 Departament of Medical Clinic, Medical School of Ribeirão Preto, University of São Paulo, USP, Ribeirão Preto, SP Brazil; 8 Department of Surgery and Anatomy, School of Medicine of Ribeirão Preto, University of São Paulo, Sao Paulo, Brazil

**Keywords:** Adenoma, Adenocarcinoma, Colorectal, Genetic markers, *ETV4*

## Abstract

**Background:**

Colorectal cancer (CRC) is one of the most common cancers worldwide; it is the fourth leading cause of death in the world and the third in Brazil. Mutations in the *APC, DCC, KRAS* and *TP53* genes have been associated with the progression of sporadic CRC, occurring at defined pathological stages of the tumor progression and consequently modulating several genes in the corresponding signaling pathways. Therefore, the identification of gene signatures that occur at each stage during the CRC progression is critical and can present an impact on the diagnosis and prognosis of the patient. In this study, our main goal was to determine these signatures, by evaluating the gene expression of paired colorectal adenoma and adenocarcinoma samples to identify novel genetic markers in association to the adenoma-adenocarcinoma stage transition.

**Methods:**

Ten paired adenoma and adenocarcinoma colorectal samples were subjected to microarray gene expression analysis. In addition, mutations in *APC, KRAS* and *TP53* genes were investigated by DNA sequencing in paired samples of adenoma, adenocarcinoma, normal tissue, and peripheral blood from ten patients.

**Results:**

Gene expression analysis revealed a signature of 689 differentially expressed genes (DEG) (fold-change> 2, *p*< 0.05), between the adenoma and adenocarcinoma paired samples analyzed. Gene pathway analysis using the 689 DEG identified important cancer pathways such as remodeling of the extracellular matrix and epithelial-mesenchymal transition. Among these DEG, the *ETV4* stood out as one of the most expressed in the adenocarcinoma samples, further confirmed in the adenocarcinoma set of samples from the TCGA database. Subsequent in vitro siRNA assays against *ETV4* resulted in the decrease of cell proliferation, colony formation and cell migration in the HT29 and SW480 colorectal cell lines. DNA sequencing analysis revealed *KRAS* and *TP53* gene pathogenic mutations, exclusively in the adenocarcinomas samples.

**Conclusion:**

Our study identified a set of genes with high potential to be used as biomarkers in CRC, with a special emphasis on the *ETV4* gene, which demonstrated involvement in proliferation and migration.

**Supplementary Information:**

The online version contains supplementary material available at 10.1186/s12885-021-07857-x.

## Background

Colorectal cancer (CRC) is the third most common cancer worldwide. It represents 10.6 and 9.4% of the total number of cancers in men and women, respectively [1]. Among the different causes of CRC, approximately 75% appears sporadically, that is, without any apparent etiological predisposition [2]. Most sporadic CRC (80%) develops from tubular/villous adenoma [3, 4], and approximately 15% develops from serrated polyps [5]. These tumors, although rare, can also arise from M-cells of the gut-associated lymphoid tissue (GALT) [6–9].

The frequency of transformation of an advanced adenoma into a carcinoma can range from 2.6 to 5.6% depending on the age of the patients [10]. Although this frequency is apparently low, CRC was responsible for 935.173 deaths worldwide and represented about 1.931.590 new cases in 2020 [1]. Despite the high incidence of CRC, and the extensive molecular profiling of these tumors, there are still no available molecular markers that can predict the progression from adenoma to adenocarcinoma.

The goal of this study was to identify a gene signature able to discriminate between adenoma and adenocarcinoma and potentially identify novel CRC biomarkers. To achieve our goal, we performed gene expression profiling in ten paired colorectal adenomas and adenocarcinomas samples. Among the differentially expressed genes (DEG), the *ETV4* gene*,* a variant transcription factor of the ETS family, showed high upregulated expression in the adenocarcinoma samples as compared to adenoma. The overexpression of this gene was further confirmed in the TCGA databases adenocarcinoma samples. Moreover, we observed that the ETV4 knockdown led to decrease in cell proliferation and migration on CRC cell lines, suggesting its potential role in CRC tumorigenesis. Finally, DNA sequencing analysis of the paired samples revealed mutations in the *KRAS* and *TP53* genes in the adenocarcinomas.

## Materials and methods

### Samples

Paired adenoma, adenocarcinoma and adjacent normal tissue samples from 10 patients were obtained during the surgical procedure at the Clinical Hospital of the Faculty of Medicine, University of São Paulo, Ribeirão Preto (HC-FMRP/USP) under the approval of the HC-FMRP/USP Research Ethics Committee (No 12636/2010). Initially, during the colonoscopy exam, adenomas were collected from patients who also had a second lesion with a clinical characteristic of adenocarcinoma and surgical indication. Each adenoma specimen was divided into two parts: one part was sent for histopathological analysis and the other part was frozen immediately after collection in liquid nitrogen. Patients who underwent the surgical procedure had the tumor resected including a safety margin. Fragments of the tumor and from the margin were also removed and divided in half; one-half was placed in formalin for histopathological analysis and the other half was immediately frozen in liquid nitrogen, still in the operating room. Only patients who had histopathological confirmation for the simultaneous presence of adenoma, adenocarcinoma and non-tumor tissue, were selected for molecular analysis. During the collections, all the polyps found were only within adenomas.

Additionally, fifteen adenomas and six adenocarcinoma samples were collected to validate the microarray gene expression profiling by RT-qPCR. The fifteen adenoma samples were isolated from seven males and eight females, with an average age and SD of 66.4±8.683 years (range 48–79). The six adenocarcinoma samples were isolated from five males and one female, with an average age and SD of 74.3±8.500 years (range 62–87). Adenoma samples were collected by colonoscopy and adenocarcinoma samples, by surgery. Using the same methodology used in the matched samples, half of each fragment was sent for histopathological analysis and the other half was frozen in liquid nitrogen for subsequent DNA and RNA isolation.

The inclusion criteria for the selection of samples were based on the clinical and histopathological diagnosis and it included: positive diagnosis for colorectal cancer and presence of adenoma and the agreement to participate in the study, by a signed consent form. Exclusion criteria were: previous treatment (chemo or radiotherapy) and history of Familial Adenomatous Polyposis (FAP), Hereditary Non-Polyposis Syndrome (HNPCC) or inflammatory bowel disease. Clinical data from the patients are shown in Table S1, Additional File 1.

### DNA and RNA isolation

Total RNA and DNA were isolated from frozen tissue using TRIzol® Reagent (Invitrogen) according to the manufacturer’s instructions (DNAse and RNAse were used, respectively). The concentrations were evaluated in a NanoVue Plus® spectrophotometer (GE Life Sciences). Specifically for the samples used in the microarrays, the RNA was isolated with RNeasy kit (Qiagen) and its quality assessed by the 2100 Bioanalyzer equipment (Agilent Technologies). Only samples that showed a RIN (RNA Integrity Number) greater than or equal to seven, were considered. The remaining 22 samples, used to validate the microarray results by RT-qPCR, had their RNA integrity assessed on 1.5% agarose gel stained with ethidium bromide.

### Mutation screening using high resolution melting (HRM) assay

Adenoma and adenocarcinoma samples were subjected to mutational screening using the HRM assay for the entire *APC* and *TP53* gene-coding regions. These regions were amplified with specific sense and antisense primers that flanked each intron/exon, as previously described by Miyoshi et al. (1992) [11] and Bastien et al. (2008) [12] respectively. The HRM analysis was performed in the 7500 Fast Real-Time PCR System (Applied Biosystems), using MeltDoctor HRM Master Mix (Applied Biosystems), according to manufacturer’s instructions.

### DNA sequencing

DNA fragments amplified by PCR-HRM, showing abnormal melting curves by the HRM assay were subjected to direct sequencing in an automatic capillary sequencing system ABI 3500 XL (Applied Biosystems), using BigDye Terminator kit, following the manufacturer’s instructions. The same methodology was used to sequence exon 2 of the *KRAS* gene, using specific sense and antisense primers that flanked each intron/exon, as previously described at Fassina et al. (2010) [13]. The sequencing results were analyzed in Chromas Lite v2.1 [14].

The normal paired tissue of the mutated samples was also sequenced, to investigate whether the mutations were of somatic or germline origin. The sequences obtained were compared to the reference from the GenBank NM_000038.5, GenBank NM_000546.5 and GenBank NM_004985.4, respectively to APC, TP53 and KRAS genes.

Pathogenicity prediction was performed in Sift [15] and Mutation Taster [16] online tools and only mutations that were predicted as damaging in both tools were classified as pathogenic.

### Microarray hybridizations

To investigate differential gene expression between colorectal adenoma and adenocarcinoma, the platform Whole Human Genome Microarray Kit 4x44K v2 (G4112F, Agilent Technologies) was employed. Prior to the hybridizations, 200 ng of total RNA from each sample were used for cDNA synthesis. The arrays slides were washed following the manufacturer’s guidelines and then scanned using the GenePix 4000B scanner (Axon Instruments) with the GenePix Pro 6.0 software and the hybridization signal intensity of each array was extracted using the Agilent Feature Extraction software 9.5.3.1. (Agilent Technologies).

### Microarray data analysis

To evaluate the data quality, we used the array QualityMetrics R/Bioconductor package [17–19]. Normalization was performed by a three-step approach with the R/Bioconductor limma package methods [17, 18, 20]. Initially it was applied as a cyclic loss method between technical replicates, quantile between samples of adenoma and adenocarcinoma group and quantile between arrays [21]. Then, the detection of differentially expressed genes (DEG) was also performed by the limma package, applying the Benjamini-Hochberg method for *p*-value correction [20, 22]. To evaluate the expression pattern of DEG, Euclidian distance and complete linkage was performed for genes and samples clustering, and then visualized in a heatmap.

### Gene expression validation

The reverse transcription reaction was performed using High Capacity cDNA Reverse Transcription Kit (Applied Biosystems) according to the manufacturer’s instructions. After synthesis, the cDNA was diluted 1:5 and then used in quantitative PCR.

TaqMan probes (Applied Biosystems and IDT) were used for RT-qPCR validation of the genes previously selected by microarray. To avoid amplification of genomic DNA (gDNA), no-RT negative control and Taqman probes in exon-exon junction were used. The *HPRT1* (4326321E, Applied Biosystems) housekeeping gene was chosen as endogenous control. Primers and probes for gene expression were *IL-6* (Hs00174131, Applied Biosystems), *IL-8* (Hs00174103, Applied Biosystems), *OSM* (Hs00171165_M1, Applied Biosystems), *SFRP4* (Hs.PT.51.1726538.g, IDT), *ETV4* (Hs.PT.56a.23047301.g, IDT), *SIM2* (Hs.PT.51.20479148.g, IDT), *ESM1* (Hs.PT.51.19279572.g, IDT) e *RETNLB* (Hs. PT.51.1296566, IDT).

All reactions were performed in an ABI Prism® 7500 Fast Sequence Detection System (Applied Biosystems). The relative expression for each gene was calculated by the 2^-ΔΔCT^ method [23].

### TCGA data analysis

The Hiseq platform gene expression level 3 RNASeqV2 data from normal and tumor samples from Colon Adenocarcinoma (COAD – normal - 41 and tumor - 285) and Rectal Adenocarcinoma (READ - normal - 10 and tumor - 94) were downloaded from the database The Cancer Genome Atlas (TCGA), on March 22, 2017, through the TCGAbiolinks R/Bioconductor package [24]. Differential expression analysis was performed using EdgeR R/Bioconductor package [25]. We considered DEG absolute values of log2 fold-change> 1 and *p*-value adjusted by FDR≤0.05.

### Gene pathways

Gene pathway analysis was performed using the 689 DEG in the MetaCore from Clarivate Analytics. For the analysis in MetaCore we used the fold change of each gene to obtain the enriched gene pathways.

### Culture and siRNA assay

For functional assays, HT29 and SW480 colorectal carcinoma cell lines were used (cell lines kindly provided by Prof. Eloiza Helena Tajara da Silva, from UNESP - University of São Paulo State). The cell lines were cultivated in RPMI (Roswell Park Memorial Institute) 1640 medium (Gibco) supplemented with 10% fetal bovine serum (FBS) and 0.5% penicillin-streptomycin under controlled temperature and humidity conditions (37 °C, 5% CO2, 95% humidity). For siRNA inhibition studies, cells were transfected with *ETV4* siRNA (si*ETV4*) or negative control (siCTRL) (Sigma-Aldrich, St. Louis, MO) in a final concentration of 30 nM, using Lipofectamine RNAiMAX reagent (Invitrogen, Carlsbad, CA, USA), according to manufacturer’s instructions. After 48 h of transfection, cells were collected for functional assays, which were all performed in triplicates.

### Cell proliferation assay

Cell proliferation assay was performed using CFSE (5,6-carboxyfluorescein diacetate succinimidyl ester) according to the manufacturer’s instructions. Cells were transfected (siCTRL and si*ETV4*) and labeled with CFSE simultaneously. A third group of cells with no treatment was used for the cytometer calibration. After 24 h of labeling, cells were evaluated in a FACSCalibur flow cytometer (Becton Dickinson, Franklin Lakes, NJ, USA), and consecutively analyzed every 24 h for a total of 96 h.

### Anchorage-dependent colony formation assay

Colorectal cancer cell lines were transfected and cultured in a density of 500 cells/well. After 12 days of culture, the cells were washed with PBS, fixed with 4% paraformaldehyde, and stained with 0.5% crystal violet. The plates were then photographed on ImageQuant LAS 4000 (GE Healthcare) and colonies were counted.

### Transwell migration assay

Cell migration assays were performed on 24-well plates with 8 μm transwell inserts (Greiner Bio One). After 48 h of transfection, 1 _×_ 10^5^ cells were seeded on top of the insert in 200 μl of serum-free medium. In the bottom of the well, cells were seeded in 600 μl of 10% FBS medium. After 24 h of migration, cells were fixed, stained with 0.5% crystal violet and the non-migrating cells from the top of the insert were cleaned with cotton swabs. The inserts were then photographed on ImageQuant LAS 4000 (GE Healthcare) and cells were manually counted.

### Statistical analysis

Statistical analysis was carried out in the GraphPad Prism 6.0 for Windows (GraphPad Software, San Diego, California, USA) [26]. Mann-Whitney’s test was applied for comparisons between two-independent groups. Statistical analysis of the proliferation assay was determined by two-way ANOVA followed by Bonferroni multiple comparisons test. *P* values ≤ 0.05 were considered significant.

## Results

### DNA sequencing and gene expression analysis of paired samples of colorectal adenoma and adenocarcinoma

DNA sequencing analysis of the ten adenoma-adenocarcinoma sample set, revealed seven mutations in the *APC* gene, six synonymous and one non-synonymous mutation, all of them germline polymorphisms. Both *TP53* and *KRAS* genes showed three and two somatic damaging mutations (P152L/R273C/R273H and G12A/G2D), in the ten paired samples, respectively. In addition, two polymorphisms were observed in the *TP53* gene (Table S2, Additional File 2). Simultaneous mutations co-occurring in the three genes were observed only in three of the ten adenoma/adenocarcinoma samples.

**Fig. 1:**
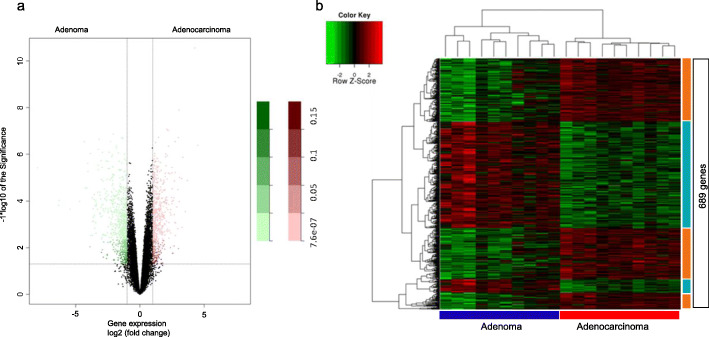
Microarray gene expression analysis in the 10 paired colorectal adenoma and adenocarcinoma samples of this study. *A:* Volcano Plot representing the set of genes analyzed by gene expression in the ten paired samples of colorectal adenoma and adenocarcinoma. Pink dots represent upregulated genes in adenocarcinoma when compared to adenoma; Green dots represent upregulated genes in adenoma when compared to adenocarcinoma. *B:* Unsupervised hierarchical clustering analysis in adenoma and adenocarcinoma samples based on the 689 differentially expressed genes (log 2 FC≥2 and FDR≤0,05)

Gene expression analysis from the ten matched colorectal adenoma-adenocarcinoma identified 689 differentially expressed genes (DEG) between these tissue types: 329 genes were upregulated in adenocarcinomas and 360 upregulated in adenomas (Fig. 1a). Unsupervised hierarchical clustering analysis of the 689 DEG was able to delimitate gene clusters specific for each tissue (Fig. 1b).

Although we observed some non-synonymous mutations in the *TP53* and *KRAS* genes, those genes were not differentially expressed in our microarray analyses.

The 689 DEGs were used to evaluate the enrichment of gene pathways potentially related to the adenoma-adenocarcinoma transition. Of the ten pathways with the highest number of genes, the pathways of cell adhesion and remodeling, epithelial-mesenchymal transition and the IGF family pathway (related to CRC) stood out.

In order to select genes with a potential role in the adenoma-adenocarcinoma transition process, we applied two filters: first the top 50 most DEG between colorectal adenoma and adenocarcinoma samples (Table 1) were selected. A second filter selected the top 50 genes DEG across all the ten adenocarcinoma-adenoma paired samples (Table 2).

**Table 1:** Top 50 DEG genes, 25 upregulated and 25 downregulated, between the paired colorectal adenocarcinoma and adenoma samples (presented by FC)

Gene	RefSeq	Description	FC^a^	FDR^b^
**Upregulated**				
***SFRP4***	**NM_003014.3**	**secreted frizzled-related protein 4**	**4.51**	**0.00072**
***SIM2***	**NM_005069.3**	**single-minded family bHLH transcription factor 2**	**4.26**	**7.63E-07**
*EREG*	NM_001432.2	epiregulin	3.54	0.01111
*FAP*	NM_001291807.1	fibroblast activation protein, alpha	3.34	0.00112
*COL11A1*	NM_001190709.1	collagen, type XI, alpha 1	3.09	0.00228
*FUT1*	NM_000148.3	fucosyltransferase 1	3.04	0.00017
***ESM1***	**NM_001135604.1**	**endothelial cell-specific molecule 1**	**2.98**	**0.00060**
*FABP6*	NM_001040442.1	fatty acid binding protein 6, ileal	2.95	0.00420
***IL8***	**NM_000584.3**	**interleukin 8**	**2.94**	**0.02829**
***OSM***	**NM_020530.4**	**oncostatin**	**2.87**	**0.00913**
***IL6***	**NM_000600.3**	**interleukin 6**	**2.76**	**0.03991**
*COL1A1*	NM_000088.3	collagen, type I, alpha 1	2.75	0.00181
*STC2*	NM_003714.2	stanniocalcin 2	2.74	0.00060
*TDO2*	NM_005651.3	tryptophan 2,3-dioxygenase	2.69	0.00264
*OTX1*	NM_001199770.1	orthodenticle homeobox 1	2.68	0.01271
*WNT2*	NM_003391.2	wingless-type MMTV integration site family member 2	2.65	0.00601
*CTHRC1*	NM_001256099.1	collagen triple helix repeat containing 1	2.51	0.00401
*COL8A1*	NM_001850.4	collagen, type VIII, alpha 1	2.50	0.00706
*NOX4*	NM_001143836.2	NADPH oxidase 4	2.49	0.00154
*TNFAIP6*	NM_007115.3	tumor necrosis factor, alpha-induced protein 6	2.48	0.00776
*INHBA*	NM_002192.2	inhibin, beta A	2.47	0.00594
*CYR61*	NM_001554.4	cysteine-rich, angiogenic inducer, 61	2.41	0.01300
*GREM1*	NM_001191322.1	gremlin 1, DAN family BMP antagonist	2.40	0.03523
*CXCL10*	NM_001565.3	chemokine (C-X-C motif) ligand 10	2.40	0.01026
***ETV4***	**NM_001079675.2**	**ets variant 4**	**2.19**	**0.00060**
**Downregulated**				
*CLCA1*	NM_001285.3	chloride channel accessory 1	−7.97	0.00155
*DEFA5*	NM_021010.1	defensin, alpha 5, Paneth cell-specific	−6.31	0.00844
*ITLN1*	NM_017625.2	intelectin 1	−6.29	0.00668
*ZG16*	NM_152338.3	zymogen granule protein 16	−5.90	0.01116
*DEFA6*	NM_001926.3	defensin, alpha 6, Paneth cell-specific	−5.89	0.01083
*FCGBP*	NM_003890.2	Fc fragment of IgG binding protein	−4.68	0.00706
***RETNLB***	**NM_032579.2**	**resistin like beta**	**−4.33**	**0.00060**
*ITLN2*	NM_080878.2	intelectin 2	−4.26	0.00391
*FAM55D*	NM_001077639.1	neurexophilin and PC-esterase domain family, member 4	−4.12	0.00392
*HEPACAM2*	NM_001039372.2	HEPACAM family member 2	−4.05	0.00131
*B3GNT6*	NM_138706.4	UDP-GlcNAc:betaGal beta-1,3-N-acetylglucosaminyltransferase 6	−4.03	0.00177
*BEST2*	NM_017682.2	bestrophin 2	−3.86	0.00099
*REG4*	NM_001159352.1	regenerating islet-derived family member 4	−3.81	0.04009
*UGT2B17*	NM_001077.3	UDP glucuronosyltransferase 2 family, polypeptide B17	−3.71	0.00601
*SPINK4*	NM_014471.1	serine peptidase inhibitor, Kazal type 4	−3.68	0.03954
*DPP10-AS1*	NR_036580.1	DPP10 antisense RNA 1	−3.67	0.01926
*SLC4A4*	NM_001098484.2	solute carrier family 4	−3.63	0.01004
*UGT2B15*	NM_001076.3	UDP glucuronosyltransferase 2 family polypeptide B15	−3.56	0.00500
*KLK12*	NM_019598.2	kallikrein-related peptidase 12	−3.56	0.00633
*L1TD1*	NM_001164835.1	LINE-1 type transposase domain containing 1	−3.52	0.02738
*ADH1A*	NM_000667.3	alcohol dehydrogenase 1A (class I) alpha polypeptide	−3.48	0.00852
*PLA2G2A*	NM_000300.3	phospholipase A2, group IIA (platelets synovial fluid)	−3.43	0.01981
*VSIG2*	NM_014312.3	V-set and immunoglobulin domain containing 2	−3.43	0.00681
*ADH1C*	NM_000669.4	alcohol dehydrogenase 1C (class I), gamma polypeptide	−3.39	0.00776
*MB*	NM_005368.2	myoglobin	−3.25	0.00144

^a^FC (log2 fold change); ^b^FDR (false discovery rate). The genes in bold were selected for validation by RT-qPCR

**Table 2:** Top 50 DEG, 29 upregulated and 21 downregulated, observed exclusively in all the ten samples of adenocarcinoma (presented by FC)

Gene	RefSeq	Description	FC^a^	FDR^b^
**Upregulated**				
*SIM2*	NM_005069.3	single-minded family bHLH transcription factor 2	4.26	7.63E-07
*FAP*	NM_001291807.1	fibroblast activation protein, alpha	3.34	0.00112
*FUT1*	NM_000148.3	fucosyltransferase 1	3.04	0.00017
*ESM1*	NM_001135604.1	endothelial cell-specific molecule 1	2.98	0.00060
*FABP6*	NM_001040442.1	fatty acid binding protein 6, ileal	2.95	0.00420
*COL1A1*	NM_000088.3	collagen, type I, alpha 1	2.75	0.00181
*WNT2*	NM_003391.2	wingless-type MMTV integration site family member 2	2.65	0.00601
*ELN*	NM_000501.3	elastin	2.22	0.00072
*ETV4*	NM_001079675.2	ets variant 4	2.18	0.00060
*ACAN*	NM_001135.3	aggrecan	2.12	0.00107
*LARP6*	NM_001286679.1	La ribonucleoprotein domain family, member 6	2.00	0.00111
*FADS1*	NM_013402.4	fatty acid desaturase	2.00	0.00099
*SCRN1*	NM_001145513.1	secernin 1	1.97	0.00188
*LY6E*	NM_001127213.1	lymphocyte antigen 6 complex, locus E	1.95	0.00691
*TCFL5*	NM_006602.2	transcription factor-like 5 (basic helix-loop-helix)	1.91	0.00426
*PROCR*	NM_006404.4	protein C receptor, endothelial	1.90	0.00745
*FAM150A*	NM_001195732.1	family with sequence similarity 150 member A	1.87	0.00283
*COL5A2*	NM_000393.3	collagen, type V, alpha 2	1.73	0.00601
*FAM72D*	NM_207418.2	family with sequence similarity 72 member D	1.62	0.00262
*SPARC*	NM_003118.3	secreted protein, acidic, cysteine-rich (osteonectin)	1.62	0.00420
*PARVB*	NM_001003828.2	parvin, beta	1.56	0.01610
*VEGFA*	NM_001025366.2	vascular endothelial growth factor A	1.46	0.00123
*UBE2C*	NM_001281741.1	ubiquitin-conjugating enzyme	1.45	0.00060
*AUNIP*	NM_001287490.1	aurora kinase A and ninein interacting protein	1.35	0.00177
*GK*	NM_000167.5	glycerol kinase	1.26	0.00437
*LRP8*	NM_001018054.2	low density lipoprotein receptor-related protein 8, apolipoprotein e receptor	1.15	0.01137
*DIAPH3*	NM_001042517.1	diaphanous-related formin 3	1.11	0.00178
*FAM162B*	NM_001085480.2	family with sequence similarity 162 member B	1.05	0.04851
*CENPJ*	NM_018451.4	centromere protein J	1.04	0.00243
**Downregulated**				
*CLCA1*	NM_001285.3	chloride channel accessory 1	−7.97	0.00155
*HEPACAM2*	NM_001039372.2	HEPACAM family member 2	−4.05	0.00131
*B3GNT6*	NM_138706.4	UDP-GlcNAc:betaGal beta-1,3-N-acetylglucosaminyltransferase 6	−4.03	0.00177
*BEST2*	NM_017682.2	bestrophin 2	−3.86	0.00099
*L1TD1*	NM_001164835.1	LINE-1 type transposase domain containing 1	−3.52	0.02738
*ADH1A*	NM_000667.3	alcohol dehydrogenase 1A (class I) alpha polypeptide	−3.48	0.00852
*ADH1C*	NM_000669.4	alcohol dehydrogenase 1C (class I), gamma polypeptide	−3.39	0.00776
*GALNT8*	NM_017417.1	polypeptide N-acetylgalactosaminyltransferase 8	−3.24	0.00084
*SPTLC3*	NM_018327.2	serine palmitoyltransferase, long chain base subunit 3	−2.99	0.00244
*SPINK2*	NM_001271718.1	serine peptidase inhibitor, Kazal type 2 (acrosin-trypsin inhibitor)	−2.49	0.01166
*ATOH8*	NM_153778.3	atonal homolog 8	−2.32	0.00643
*KCNMA1*	NM_001014797.2	potassium large conductance calcium-activated channel, subfamily M, alpha member 1	−2.27	0.00060
*NEURL*	NM_004210.4	neuralized E3 ubiquitin protein ligase 1	−2.19	0.00109
*PIGR*	NM_002644.3	polymeric immunoglobulin receptor	−2.14	0.01272
*MFSD4*	NM_181644.4	major facilitator superfamily domain containing 4	−2.11	0.00201
*ARHGAP44*	NM_014859.4	Rho GTPase activating protein 44	−2.06	0.00060
*SERTAD4*	NM_019605.3	SERTA domain containing 4	−1.82	0.00072
*PLA2G10*	NM_003561.1	phospholipase A2, group X	−1.73	0.04438
*NEDD4L*	NM_001144964.1	eural precursor cell expressed, developmentally down-regulated 4-like, E3 ubiquitin protein ligase	−1.61	0.00123
*ACADVL*	NM_000018.3	acyl-CoA dehydrogenase, very long chain	−1.20	0.00099
*PID1*	NM_001100818.1	phosphotyrosine interaction domain containing 1	−1.19	0.01212

^a^FC (log2 fold change); ^b^FDR (false discovery rate). The genes in bold are the same ones selected in Table 1 for validation by RT-qPCR

**Fig. 2:**
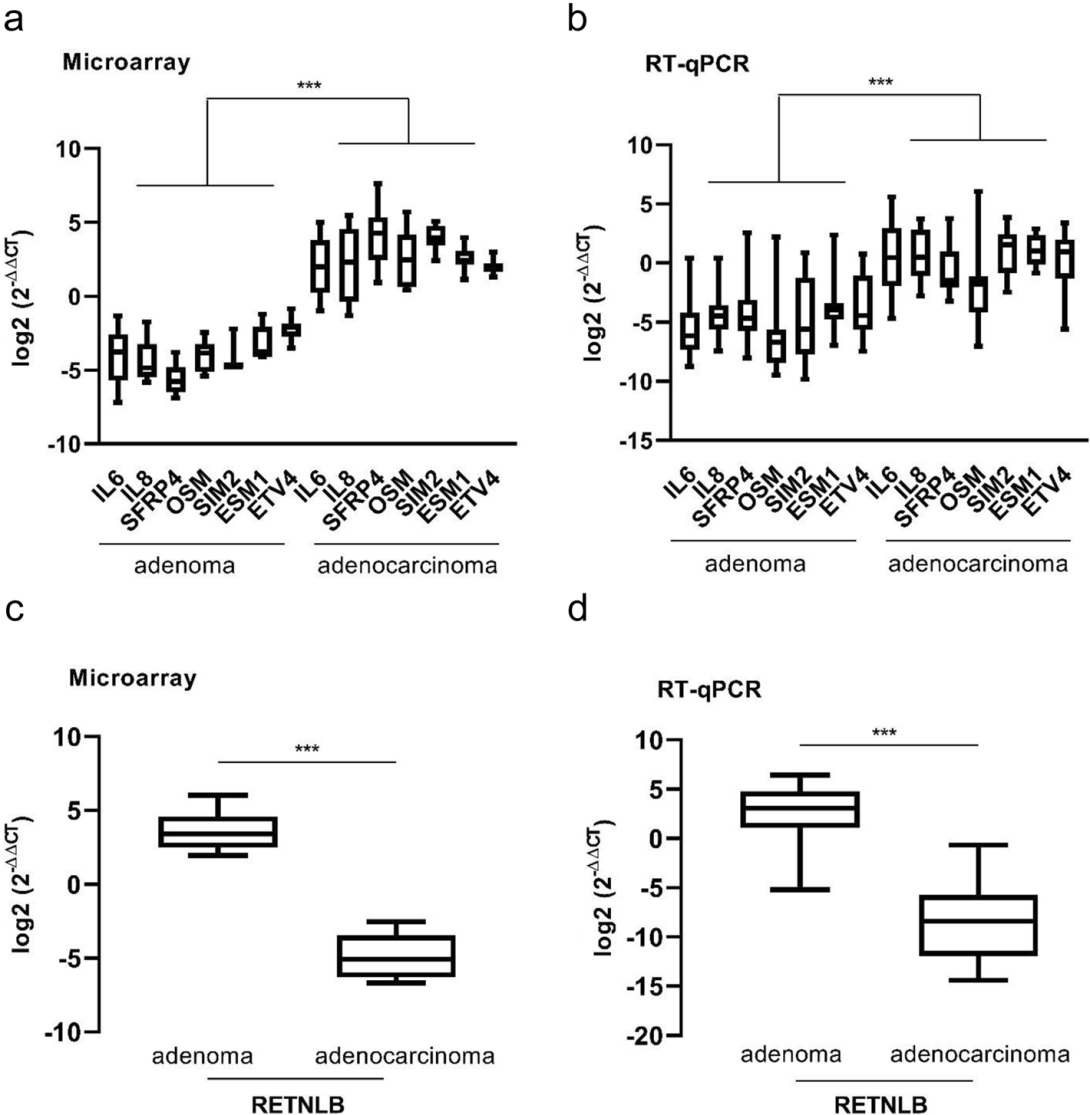
Validation of microarray data by RT-qPCR. **a** Microarray data of the 10 paired adenoma/adenocarcinoma paired samples, showing the upregulation of seven genes in adenocarcinomas compared to adenomas. **b** RT-qPCR results showing the validation of the microarray results of the same set of genes in a larger set of samples (25 adenoma and 16 adenocarcinoma samples, of which 10 adenoma/adenocarcinoma paired samples and 15 adenoma and 6 adenocarcinoma additional new samples). **c** and **d** Relative expression of the RETNLB gene found downregulated in adenocarcinomas when compared to adenomas by microarray (10 paired adenoma-adenocarcinoma samples) and RT-qPCR (10 adenoma/adenocarcinoma paired samples plus 15 adenoma and 6 adenocarcinoma samples) analysis, respectively. Fold changes (log2 FC) between the expression means in adenomas and adenocarcinomas were A = 6.75, B=4.93, C=8.50 and D=11.12. Mann-Whitney’s test was used for statistical analysis (A and B: ***p < 0.0006; C and D: ***p < 0.0001)

To confirm the gene expression data obtained from microarray analysis, eight genes were selected for validation by RT-qPCR (*SIM2, ESM1, SFRP4, IL8, IL6, OSM, ETV4 and RETNLB*) on 25 adenomas and 16 adenocarcinomas (the initial 10 adenoma/adenocarcinoma paired samples and additional 15 adenomas and 6 adenocarcinoma samples). The RT-qPCR results were in agreement with the microarray data. In addition, genes presented a very similar expression pattern between the adenoma-adenocarcinoma pairs in both techniques (Fig. 2a-d), reinforcing the robustness of the data.

To further validate our findings in a different cohort using non-neoplastic tissue, we performed an in silico gene expression analysis between normal colorectal tissue and both colon and rectal adenocarcinoma, using data available at TCGA.

**Fig. 3:**
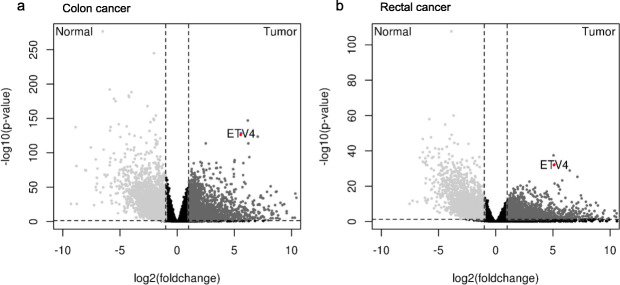
Gene expression analysis of the colon and rectum normal or cancer samples from the TCGA database. **a** Volcano plot representing the differentially expressed genes between 41 normal tissue and 285 colon adenocarcinomas samples (FDR=4.91e-129 and log2 fold change = 5.59). **b** Volcano plot representing the differentially expressed genes between 10 normal tissue samples and 94 colon adenocarcinomas samples (FDR= 5.544394e-33 and log2 fold change = 5.13). Red dot: *ETV4* gene overexpressed in cancer samples

Gene expression analyses were performed separately. First, normal colon tissue and colon adenocarcinoma were compared (Fig. 3a). A second analysis compared the normal rectal tissue with the rectal adenocarcinoma (Fig. 3b). In both analyses, *ETV4* was overexpressed in adenocarcinoma samples (Fig. 2a-b). As this gene was also found to be upregulated in our cohort (Table 1), we proceeded with a functional investigation of its role in colorectal tumorigenesis.

### *ETV4* acts in the proliferation, colony formation and cellular migration

**Fig. 4:**
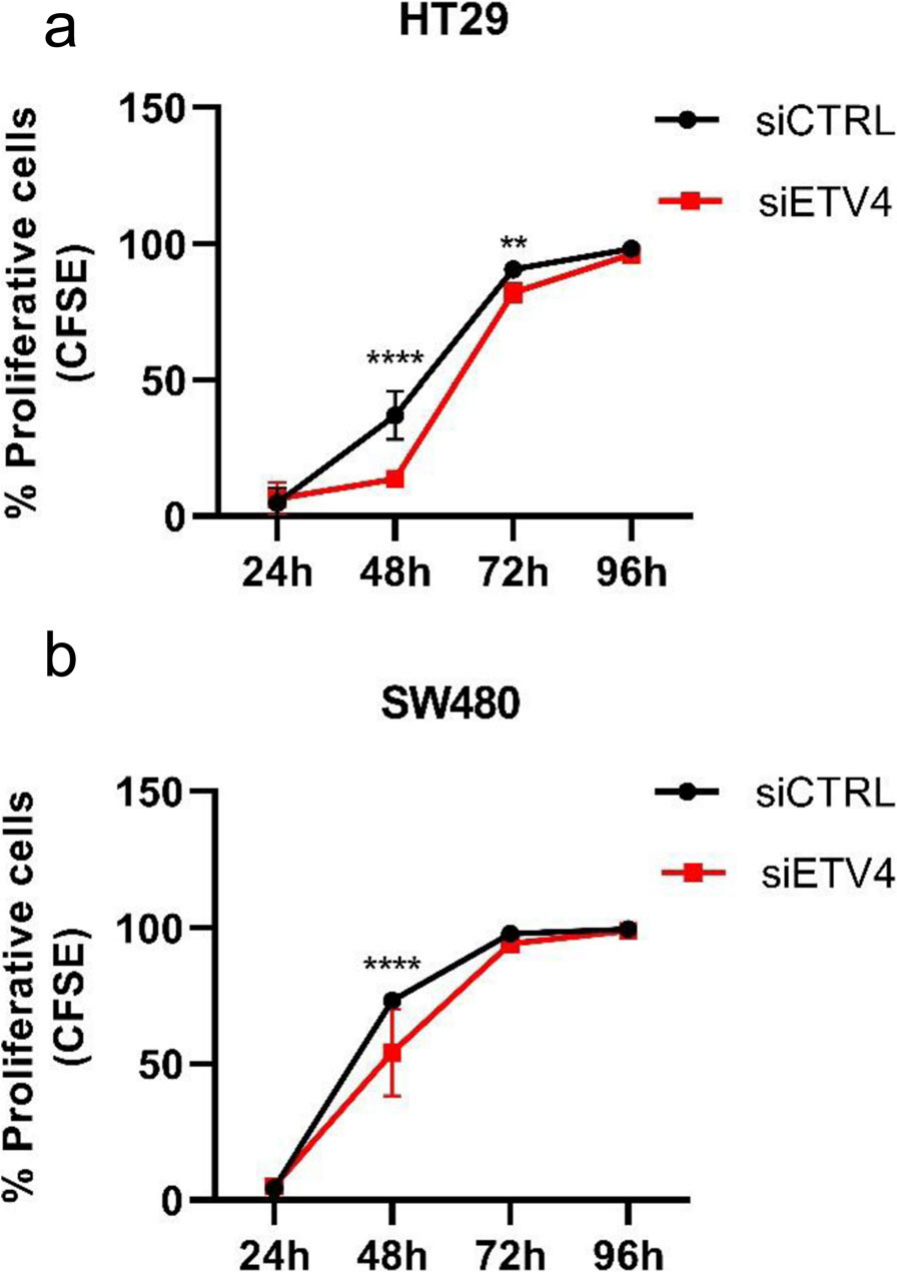
The knockdown of the *ETV4* gene led to decrease of cell proliferation in both CRC cell lines: HT29 (**a**) and SW480 (**b**). Cells were simultaneously transfected with siRNAs and stained for CFSE, and then plated in 24 well plates. Flow cytometry analysis of CFSE staining were performed every 24 h during 96 h (**p< 0.05; ****p< 0.0001 Two-way ANOVA followed by Bonferroni correction)

To investigate the possible role of *ETV4* in colorectal carcinoma tumorigenesis, HT29 and SW480 colorectal cancer cell lines were used in functional assays. Transient transfection using siRNA oligos efficiently knocked down 80% of the *ETV4* gene expression levels in HT29 cell line and 88% in SW480 cell line, as confirmed by RT-qPCR (Supplementary Figure 1, Additional File 3). Cell proliferation rates were analyzed by CFSE labelling and evaluated by flow cytometry every 24 h during 96 h. The results showed a significant reduction of HT29 cell proliferation rates at 48 h and 72 h after transfection with *ETV4*-siRNA (Fig. 4a). Accordingly, in SW480 cells depleted for *ETV4* expression, cell proliferation was significantly reduced after 48 h (Fig. 4b). Downregulation of *ETV4* expression in colorectal cell lines did not affect cell apoptosis or viability, (Supplementary Figure 2A-B, Additional File 4), indicating that the results observed are exclusively due to the modulation of cell proliferation.

**Fig. 5:**
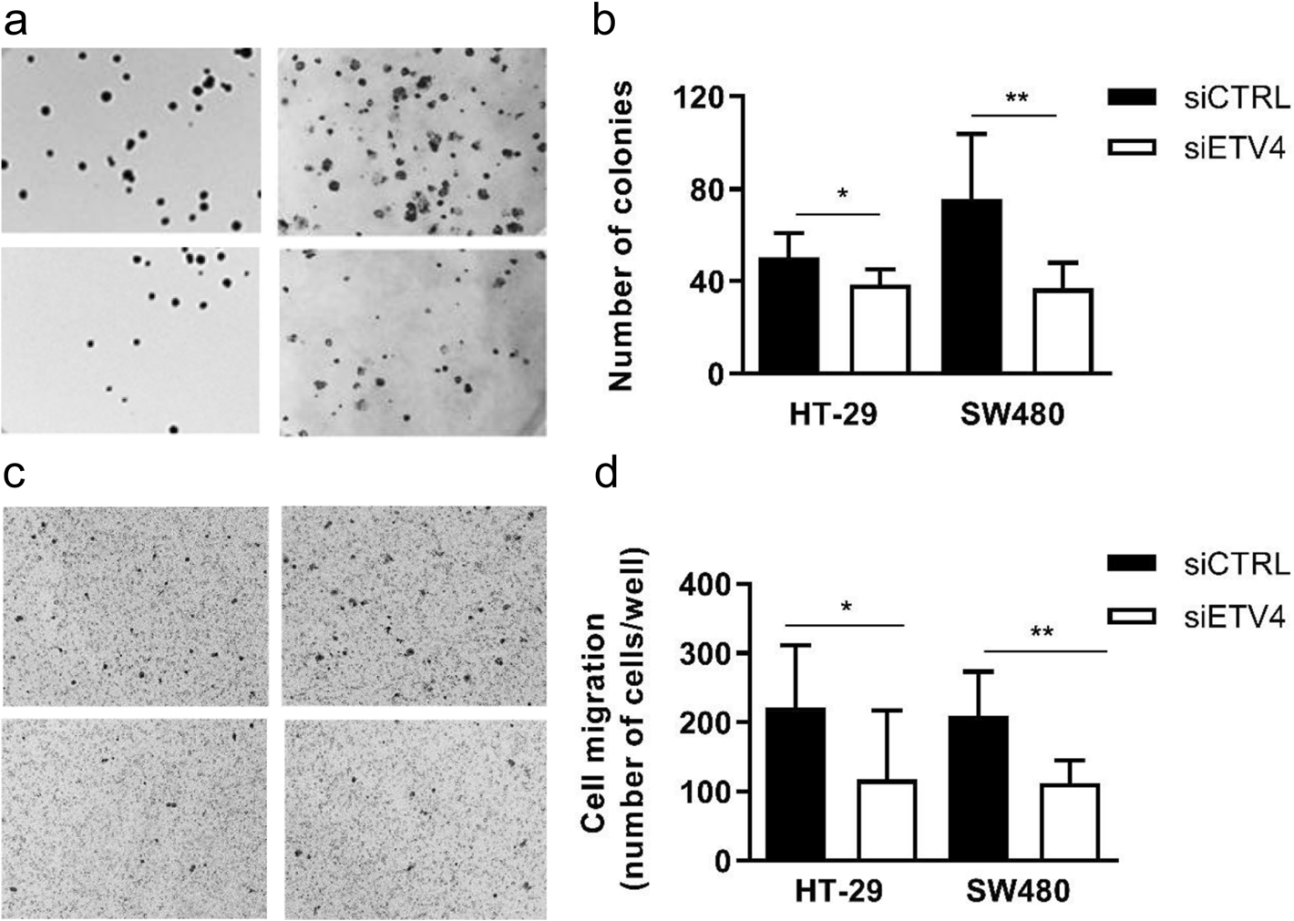
Knockdown of the *ETV4* gene reduced the clonogenic ability and cell migration in the CRC cell lines HT29 and SW480. **a** Representative images of cell colonies formed after 12 days of culture. **b** Bar graph representing the mean of three independent experiments. CRC cells were sparsely plated in 6 well plates (500 cells/well), allowed to grow for 12 days, fixed and stained for manual counting (HT29: *p*=0.0402; SW480: *p*=0.0015, Mann-Whitney statistical test). **c** Representative images of cell migration. **d** Bar graph representing the mean of three independent experiments. Cells were seeded on the top of the insert (1 _×_ 10^5^), fixed 24 h, and stained for manual counting (HT29: *p*= 0.0231; SW480: *p*=0.0152, Mann-Whitney statistical test)

Colony formation assays were also performed by counting the number of colonies formed by sparsely cultured cells after 12 days. *ETV4* gene knockdown in HT29 and SW480 cells impaired their ability to grow as colonies in 20 and 50%, respectively (Fig. 5a-b). We also evaluated the effect of *ETV4* gene knockdown on the migratory capacity of colorectal tumor cell lines. The migration assay was performed on transwell membranes 24 h after the *ETV4* knockdown in the HT29 and SW480 cell lines. As it can be seen in Fig. 5c-d, depletion of *ETV4* expression decreased cell migration in both cell lines. Taken together, these results suggest an important role of *ETV4* in diverse tumorigenic processes, such as cell proliferation, migration, and colony formation.

## Discussion

### Adenoma and adenocarcinoma colorectal gene signatures

In this study, the analysis of gene expression profiles of adenomas and adenocarcinomas by microarrays and signaling pathways analysis revealed many pathways and cellular processes associated with extracellular matrix remodeling, angiogenesis and epithelial-mesenchymal transition, as well as the IGF (Insulin-like growth factor) signaling pathway, which is known to be directly linked to colorectal cancer (Metacore from Clarivate Analytics) (Table S3, Additional File 5).

The strategy of comparing adenoma-adenocarcinoma samples from the same patient reduces sample and tumor heterogeneity, increasing the power of the study to generate a potential gene signature for the adenoma-adenocarcinoma transition.

Several studies on differentially expressed genes in CRC are found in the literature. However, the different microarray platforms and statistical methods used in these studies hamper the discovery of reliable biomarkers to be used in clinical practice. To overcome this limitation, some research groups [27–30] applied meta-analysis approaches, comparing different microarray analysis in samples from normal tissue, adenoma and adenocarcinoma or only between normal tissue and adenocarcinoma. Some of the genes described in these meta-analysis studies were also found in our data and are briefly discussed below. *FcGBP*, was found to be downregulated in our study and in normal-adenoma-carcinoma sequence according to Lee and colleagues [31]. *FcGBP* was also indicated as a prognosis marker in gallbladder cancer [32]. *CLCA1* has been described as a marker of the transition from proliferation to differentiation in CRC [33]. *CLCA1* decreased expression was also described in serum and CRC tissues, showing an inverse correlation with CRC metastasis and tumor stage [34]. *CLCA1* and *ADH1C* were shown to be downregulated in familial adenomatous polyposis [35]. S*LC4A4* associated with proliferation and migration in colon and breast cancer [36]. *COL1A1* was overexpressed in tumor tissues from colorectal adenocarcinomas and its silencing significantly inhibited proliferation, migration and invasion, while cell apoptosis was promoted [37]. *ZG16* has been associated with stemness and progression in CRC [38] and its expression has been shown to be sequentially reduced from normal tissue to adenoma and to carcinoma [39]. *ETV4* and *FABP6* were co-expressed in tumor samples and significantly associated with metastasis in CRC [40]. *DEFA6* was shown to be associated with overall survival rate and is an independent prognostic marker for CRC [41]. *L1TD1* has been described as a good prognosis marker candidate in CRC, but its elevated expression has also been associated with poor prognosis in other cancer types. These distinctive roles are dependent on its interaction partners. Several co-expression partners of *L1TD1* already described in CRC have also been observed in our study, such as *SPINK4, RETNLB, CLCA1, FcGBP, HEPACAM2, ITLN1* and*, DEFA5* [42]. The common genes observed in our study with previous meta-analysis and other studies reinforces the importance of our findings.

### *ETV4* functional validation

The *ETV4* gene (E1AF/PEA3 - ets variant 4) is a transcription factor member of the ETS oncogene family that comprises a conserved amino acid sequence, the ETS domain, the DNA binding site to the ETS oncogenes [43]. Its elevated expression has been described in several types of cancer, such as breast, ovary, prostate, gastric and colorectal [44–48].

Our assays demonstrated that *ETV4* silencing in the HT29 and SW480 CRC cell lines reduced proliferation, colony formation and cell migration. Previous studies have shown the effects of *ETV4* silencing in the reduction of cell proliferation, migration and invasion, in both colon and prostate cancer cell lines, but no data on colony formation has been previously associated with *ETV4* in CRCs [49, 50]. Hence, our results suggest that *ETV4* is important for the growth of CRC cells.

The reduction in proliferation demonstrated by Hollenhorst et al (2011) [51] in prostate cancer cell lines after *ETV4* inhibition was found in combination with a decreased *MYC* gene expression, due to the direct regulation of *MYC* by *ETV4*. Our findings are in agreement with the reduced proliferation after *ETV4* inhibition however, we identified an increase in *MYC* gene expression in adenocarcinomas as compared to adenomas.

Previous studies have shown that activation of multiple matrix metalloproteinases plays an important role in tumor invasion by degradation of the extracellular matrix in colorectal cancer [52–55]. *MMP1* was identified as a direct or indirect *ETV4* target acting on the CRC progression [56]. In a study carried out in non-small cell lung cancer [57] the *ETV4-MMP1* axis was associated with a poor prognosis. A similar relationship was observed in breast cancer, but for *ETV4* and *MMP13* [58].

In our samples, only *MMP11* (stromelysin-3) was upregulated in adenocarcinoma compared with paired adenoma samples. This metalloproteinase is described in several types of cancer, acting in the proliferation, migration and invasion control [59–61]. In CRC, elevated *MMP11* expression was associated with poor prognosis and reduced survival in stage II patients [62]. Serum levels of *MMP11* were previously shown to be significantly higher in patients with lymph node metastasis and was also identified as an independent prognostic factor for 5-year mortality in CRC [63]. *MMP11* upregulation has also been related to lymph node metastasis in non-small cell lung cancer and colorectal cancer [64, 65]. Accordingly, *MMP11* was upregulated in all our cases that also had compromised lymph nodes. To our knowledge, there is no information available about the relationship between *ETV4* and *MMP11*, so one might speculate that both genes may be involved in lymph node metastasis in CRC. Indeed, *ETV4* is related to the embryonic development of different organs, but it is also closely linked to carcinogenesis, especially in metastasis development [66].

Several studies indicate the activation of MMPs by *ETV4* [58, 67, 68]. However, it is likely that this activation occurs in dependence of expression and/or functional alterations in other genes involved in the MMP/ETV4 axis.

## Conclusions

In summary, this study identified a set of differentially expressed genes in CRC, including *FcGBP, CLCA1, ADH1C, COL1A1, ZG16*, which could be strong candidates to be used as biomarkers of colorectal adenoma-adenocarcinoma progression. Among those genes, *ETV4* was further investigated and was shown to act on proliferation and migration of CRC cell lines, indicating that *ETV4* could be a robust adenocarcinoma biomarker and a potential target for gene therapy studies in CRC.

## Supplementary Information

**Additional file 1 Table S1.** Location and pathological tissue of each patient.

**Additional file 2 Table S2.** Observed mutations in *APC,TP53* and *KRAS* genes.

**Additional file 3 Figure S1.** Relative expression of the *ETV4* gene after its silencing in HT29 and SW480 CRC cell lines. The silencing was efficient with *p* < 0,0001.

**Additional file 4 Figure S2.** Apoptosis assay. The HT29 and SW480 cell lines did not show any changes in the apoptosis’ rates (A) and cell viability (B).

**Additional file 5 Table S3.** Enrichment of signaling pathways analysis.

## Data Availability

The datasets used during this study are available from the corresponding author on reasonable request.

## Abbreviations

CRC: Colorectal cancer
GALT: Gut-associated lymphoid tissue
HC-FMRP/USP: Hospital of the Faculty of Medicine of Ribeirão Preto/University of São Paulo
FAP: Familial adenomatous polyposis
HNPCC: Hereditary Non-Polyposis Syndrome
HRM: High Resolution Melting
PCR: Polymerase Chain Reaction
DEG: Differentially expressed genes
RT-qPCR: Reverse transcription quantitative real time PCR
COAD: Colon adenocarcinoma
READ: Rectal adenocarcinoma
TCGA: The cancer genome atlas
UNESP: University of São Paulo State
RPMI: Roswell Park Memorial Institute
FBS: Fetal bovine serum
siRNA: Small interfering RNA
siCTRL: Small interfering negative control
CFSE: 5(6)-carboxyfluorescein diacetate succinimidyl ester
PBS: Phosphate buffered saline
FDR: False Discovery rate
ECM: Extracellular matrix
