# Supplementary material for: ETV4 plays a role on the primary events during the adenoma-adenocarcinoma progression in colorectal cancer
Source: BMC Cancer. 2021 Mar 1;21:207. doi: 10.1186/s12885-021-07857-x (PMC7919324; doi:10.1186/s12885-021-07857-x)
Supplement: Supplementary file 1 — Additional file 1 Table S1. Location and pathological tissue of each patient. [file 12885_2021_7857_MOESM1_ESM.docx]

| **Patient** | **Age Range** | **Adenoma** | **Region** | **Adenocarcinoma** | **Region** | **Grade** |
| --- | --- | --- | --- | --- | --- | --- |
| **01** | 71-80 | Tubular with mild dysplasia | sigmoid | Tubular invasive moderately differentiated | sigmoid | T1N0 |
| **02** | >80 | Tubulo-villous with mild dysplasia | recto | Tubular invasive poorly differentiated | recto | T2N2 |
| **03** | 61-70 | Tubular with mild dysplasia | rectosigmoid | Tubular invasive moderately differentiated | rectosigmoid | T3N1 |
| **04** | >80 | Tubular with mild dysplasia | sigmoid | Tubular invasive moderately differentiated | sigmoid | T3N0 |
| **05** | 71-80 | Tubular with moderate dysplasia | ascendant | Tubular invasive moderately differentiated | ascendant | T3N1 |
| **06** | 61-70 | Tubular with moderate dysplasia | recto low | Tubular invasive moderately differentiated | recto high | ------ |
| **07** | 31-40 | Tubular with moderate dysplasia | sigmoid | Tubular invasive moderately differentiated | sigmoid | T3N1 |
| **08** | 71-80 | Tubular with mild dysplasia | recto high | Tubular Invasive well differentiated | recto high | T3N0 |
| **09** | 61-70 | Tubular with mild dysplasia | transverse | Tubular invasive moderately differentiated | transverse | T3N0 |
| **10** | 71-80 | Tubular dysplasia of low and high grade | rectosigmoid | Tubular invasive moderately differentiated | rectosigmoid | T3N2 |
