# Supplementary material for: ETV4 plays a role on the primary events during the adenoma-adenocarcinoma progression in colorectal cancer
Source: BMC Cancer. 2021 Mar 1;21:207. doi: 10.1186/s12885-021-07857-x (PMC7919324; doi:10.1186/s12885-021-07857-x)
Supplement: Supplementary file 2 — Additional file 2 Table S2. Observed mutations in APC,TP53 and KRAS genes. [file 12885_2021_7857_MOESM2_ESM.docx]

**Supplementary Table 2**: Observed mutations in *APC,TP53* and *K-RAS* genes.

| Gene | Mutation | Prediction |
| --- | --- | --- |
| APC | Y486Y | Polimorphism |
|  | Q1244Q | Polimorphism |
|  | T1493T | Polimorphism |
|  | G1678G | Polimorphism |
|  | S1756S | Polimorphism |
|  | P1960P | Polimorphism |
|  | V1822D | Polimorphism |
| TP53 | P36P | Polimorphism |
|  | P72R | Polimorphism |
|  | **P152L** | Damaging |
|  | **R273C** | Damaging |
|  | **R273H** | Damaging |
| K-RAS | **G12A** | Damaging |
|  | **G12D** | Damaging |

The screening mutation was performed in normal tissue, adenoma and adenocarcinoma samples. Only P152L, R273C, R273H, G12A and G12D were somatic mutations (in bold).
