# Supplementary figures and images for: ETV4 plays a role on the primary events during the adenoma-adenocarcinoma progression in colorectal cancer
Source: BMC Cancer. 2021 Mar 1;21:207. doi: 10.1186/s12885-021-07857-x (PMC7919324; doi:10.1186/s12885-021-07857-x)

## Slide 1
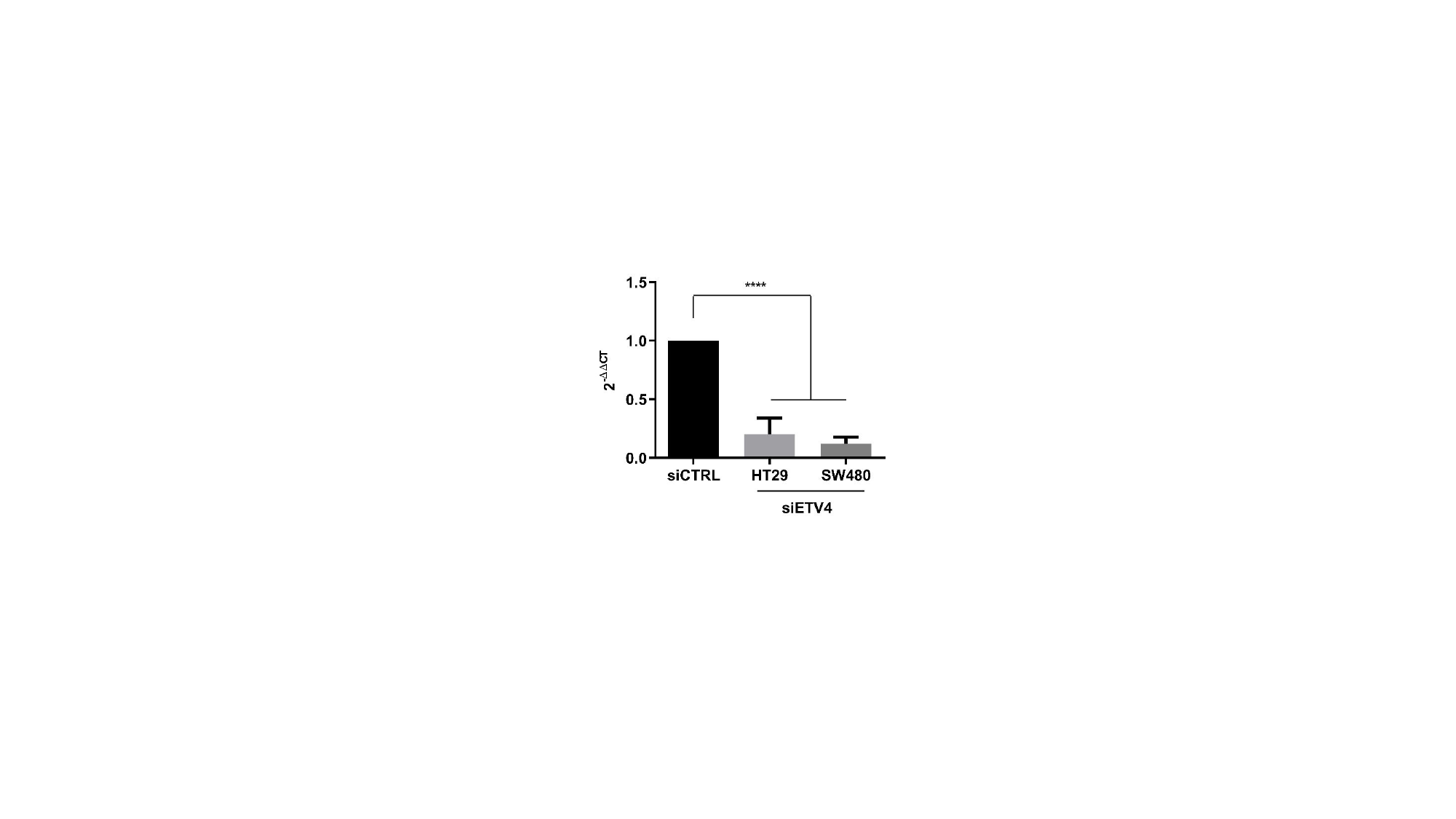

Supplement: Supplementary file 3 — Additional file 3 Figure S1. Relative expression of the ETV4 gene after its silencing in HT29 and SW480 CRC cell lines. The silencing was efficient with p < 0,0001. [file 12885_2021_7857_MOESM3_ESM.pptx]

## Slide 1
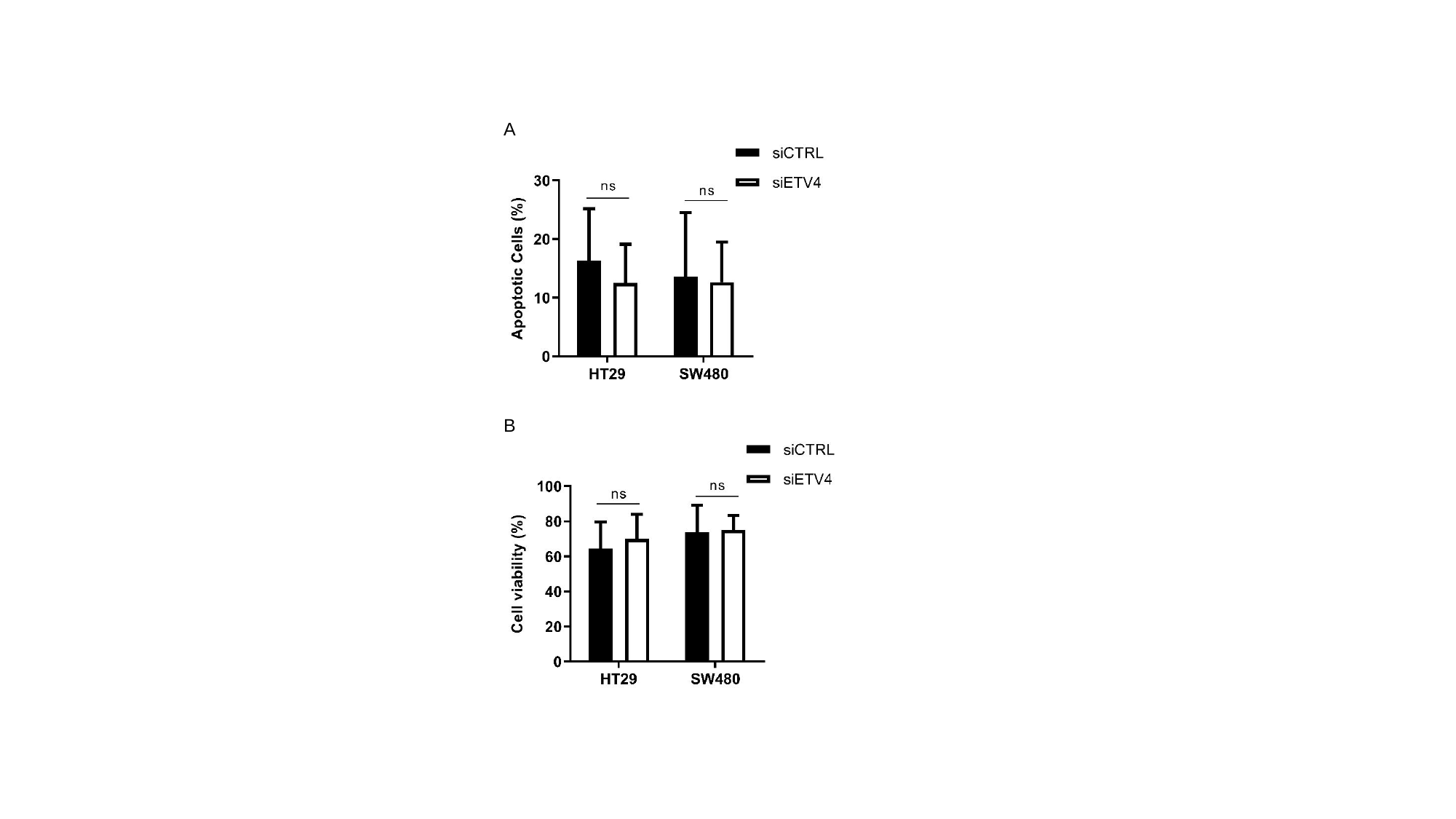

A
B

Supplement: Supplementary file 4 — Additional file 4 Figure S2. Apoptosis assay. The HT29 and SW480 cell lines did not show any changes in the apoptosis’ rates (A) and cell viability (B). [file 12885_2021_7857_MOESM4_ESM.pptx]
