# Supplementary material for: ETV4 plays a role on the primary events during the adenoma-adenocarcinoma progression in colorectal cancer
Source: BMC Cancer. 2021 Mar 1;21:207. doi: 10.1186/s12885-021-07857-x (PMC7919324; doi:10.1186/s12885-021-07857-x)
Supplement: Supplementary file 5 — Additional file 5 Table S3. Enrichment of signaling pathways analysis. [file 12885_2021_7857_MOESM5_ESM.docx]

**Supplementary Table 3:** Enrichment of signaling pathways.

| **Signaling pathways** | Genes | FC(log2) | *p* value |
| --- | --- | --- | --- |
| **Cell adhesion_ECM remodeling** | *COL1A1* | 2.754 | 0.001817 |
|  | *COL3A1* | 1.587 | 0.01629 |
|  | *COL4A3* | -1.593 | 0.031976 |
|  | *IL8* | 2.945 | 0.028296 |
|  | *KLK1* | -3.564 | 0.014367 |
|  | *KLK3* | -3.116 | 0.006334 |
|  | *SPARC* | 1.627 | 0.004202 |
|  | *PLAU* | 1.068 | 0.040273 |
|  | *SDC2* | 1.027 | 0.026623 |
|  | *VCAN* | 1.125 | 0.022739 |
| **Development_Regulation of epithelial-to-mesenchymal transition (EMT)** | *BCL2* | -1.259 | 0.002435 |
|  | *CALD1* | 1.066 | 0.0159 |
|  | *CLDN1* | 1.602 | 0.002247 |
|  | *EDNRA* | 1.319 | 0.022562 |
|  | *OSM* | 2.874 | 0.009139 |
|  | *PDGFRB* | 1.559 | 0.007177 |
|  | *TGFB2* | 1.317 | 0.003882 |
|  | *WNT* | 2.653 | 0.006011 |
| **IGF family signaling in colorectal cancer** | *CLU* | -1.467 | 0.006284 |
|  | *E2F1* | 1.358 | 0.00119 |
|  | *IBP* | 1.306 | 0.002346 |
|  | *IBP3* | 1.306 | 0.002444 |
|  | *IL8* | 2.945 | 0.028296 |
|  | *IRS1* | 1.052 | 0.043367 |
|  | *VEGFA* | 1.206 | 0.001231 |
